# Mesenchymal Stem Cell Transplantation for Liver Cell Failure: A New Direction and Option

**DOI:** 10.1155/2018/9231710

**Published:** 2018-03-04

**Authors:** Yantian Cao, Bangjie Zhang, Rong Lin, Qingzhi Wang, Jie Wang, Fangfang Shen

**Affiliations:** ^1^Department of Gastroenterology, The Third Affiliated Hospital, Xinxiang Medical University, Hua Lan Avenue, Xinxiang, Henan Province 453003, China; ^2^Department of Gastroenterology, Union Hospital of Tongji Medical College, Huazhong University of Science and Technology, 1277 Jiefang Avenue, Wuhan, Hubei Province 430022, China; ^3^M. M. School of Automation, Key Laboratory of Image Processing and Intelligent Control of Education Ministry of China, Huazhong University of Science and Technology, Wuhan, Hubei Province 430022, China; ^4^The Key Laboratory for Tumor Translational Medicine, The Third Affiliated Hospital, Xinxiang Medical University, Hua Lan Avenue, Xinxiang, Henan Province 453003, China

## Abstract

**Background and Aims:**

Mesenchymal stem cell transplantation (MSCT) became available with liver failure (LF), while the advantages of MSCs remain controversial. We aimed to assess clinical advantages of MSCT in patients with LF.

**Methods:**

Clinical researches reporting MSCT in LF patients were searched and included.

**Results:**

Nine articles (*n* = 476) related with LF patients were enrolled. After MSCT, alanine aminotransferase (ALT) baseline decreased largely at half a month (*P* < 0.05); total bilirubin (TBIL) baseline declined to a certain stable level of 78.57 *μ*mol/L at 2 and 3 months (*P* < 0.05). Notably, the decreased value (*D* value) of Model for End-Stage Liver Disease score (MELD) of acute-on-chronic liver failure (ACLF) group was higher than that of chronic liver failure (CLF) group (14.93 ± 1.24 versus 4.6 ± 5.66, *P* < 0.05). Moreover, MELD baseline of ≥20 group was a higher D value of MELD than MELD baseline of <20 group with a significant statistical difference after MSCT (*P* = 0.003).

**Conclusion:**

The early assessment of the efficacy of MSCT could be based on variations of ALT at half a month and TBIL at 2 and 3 months. And it had beneficial effects for patients with LF, especially in ACLF based on the *D* value of MELD.

## 1. Introduction

Liver failure (LF) is defined as decompensation complications performing ascites, encephalopathy, and coagulopathy of any degree, and other physiological function of liver is damaged (e.g., AST, ALT, TBIL, and ALB) [1, 2]. In the given disease courses, pathological changes, and clinical presentations, LF could be classified into three forms: acute liver failure (ALF) occurred within 48 hours to several days accompanied with many complications (infection, coagulopathy, and encephalopathy) [3]; acute-on-chronic liver failure (ACLF), with underlying chronic liver disease leading to rapid progression of liver injury, is manifested as additional jaundices and ascites [4]; and chronic liver failure (CLF) remains a course of several months or years with chronic liver diseases [5]. Of these, ACLF and CLF occur commonly. The mortality rate of them ranges from 40% to 80% [6]. In addition, current knowledge of LF pathophysiology has been limited and the therapeutic strategies of LF still not have a systematic protocol. Both physicians and surgeons were based on integrated therapy for treatment of inpatient with ALF, ACLF and CLF, which mainly included nucleoside analogs (lamivudine, telbivudine, and entecavir), glucocorticoids, plasmapheresis, and liver transplantation [7, 8]. Due to the hard-acquired complications of postoperative immunosuppression in liver transplantation, only about 5000 patients each year received solid liver transplantations [9]. Therefore, liver regeneration is still thought to be an alternative ideal therapeutic approach for LF in clinical practice via activating mature hepatocytes, endogenous stem cells and circulating stem cells for regeneration of liver cells [10].

Mesenchymal stem cells (MSCs) are characterized by differentiation, anti-inflammation and immunomodulation, and antifibrotic effect in tissue engineering [11, 12] and mainly derived from bone marrow, umbilical cord, and adipose tissue. It was not a coincidence that there were many animal researches [13–15] and clinical trials [16–18] to clarify the advantages of stem cells in liver cell failure, which achieved a good efficacy and safety. Coincidentally, our previous study also demonstrated that MSCT was considered as a promising therapeutic option for regeneration of the intestinal nerve system in gastrointestinal denervation model of murine via two aspects: directly regenerating and repairing tissue cells or indirectly activating immune cells (CD4+, regulatory T cells, etc.) to secrete immune factors (IL-2, IL-10, etc.) [11, 19]. Although our chronic inflammation model of mice induced by *Helicobacter pylori* had a risk of carcinogenesis after MSC intervention [20], the MSC effects of potential multilineage differentiation, immunomodulation, and antifibrosis hold the balance in the treatment of patients with liver cell failure. Amazingly, Okumoto et al. [21] reported that the level of stem cell factor was markedly decreased in patients with LF, and Salama [12] also reported a decrease in serum levels of the hepatic fibrosis markers (e.g., collagen matrix, PIIICP, and PIIINP). There are several mechanisms of action of MSCT in liver regeneration: endogenous stem cell activation, paracrine effect, angiogenesis, and cell fusion, in addition to actual transdifferentiation [22]. Exogenous supplement of MSCs thus may improve the liver function of patients with liver cell failure.

Here, according to the evidences of variations of ALT, TIBL, ALB, and PT, we highlight that there is an obvious effect of MSCT on the treatment of LF [1, 16, 17]. However, to date, the detailed protocols about MSCT in LF are still not discussed. Therefore, this review aimed to provide an overview of the efficacy of MSCT and to explore the optimum state of MSC treatment on liver cell failure.

## 2. Materials and Methods

### 2.1. Searching Strategies

We searched for articles published in PubMed systematically with MESH terms and text words: “stem cell,” “mesenchymal stromal cell,” “mesenchymal stem cell,” “liver failure,” and “hepatic failure.” And we enrolled all eligible articles until May 15, 2017, by screening the titles and abstracts about the MSCT in patients with LF. All clinical trials of LF treated with MSCT were included. Additionally, the reference lists of relevant articles were also scrutinized.

### 2.2. Data Selection and Extraction

All study selection and data extraction were accomplished by two investigators independently. Disagreements were resolved by a discussion. Data on the authors, publication dates, countries, participants' characteristics (e.g., number, subtypes of cells, and ways of MSC administrated), and the level of ALT, TIBL, ALB, PT, and MELD score were extracted. Trials eligible for inclusion were based on the quality of evidence included: (1) clinical trials; (2) randomized controlled trials (RCTs) and no randomized trials; (3) patients with LF; (4) therapeutic strategy at least included MSCT; and exclusion criteria included (1) duplicate publication, (2) case reports, (3) reviews, (4) animal trials, (5) no-English languages, and (6) other liver diseases except LF.

### 2.3. Quality Assessments

The Newcastle-Ottawa Scale (NOS) was adopted to assess the quality of included studies, in which 9 items to evaluate quality [11]. The total of all answers generated the final scores for each study. A high quality and a poor quality score is 5–9 and 0–4 [23].

### 2.4. Statistical Analysis

The data of ALT, TBIL, ALB, PT, and MELD score were considered as the assessment of efficacy on MSCT in patients with LF. A single article was considered as a whole to analyze. The results were expressed as mean ± standard deviation (*M* ± SD). All statistical integrations were done by using SPSS (Version 19.0) and GraphPad Prism (Version 6.0). And statistical analysis was performed by variance (ANOVA) or Student's *t*-test [19, 24]. All tests were two-tailed, and a value of *P* < 0.05 was deemed statistically significant.

## 3. Results

### 3.1. Search Results and Quality Assessment

**Figure 1:**
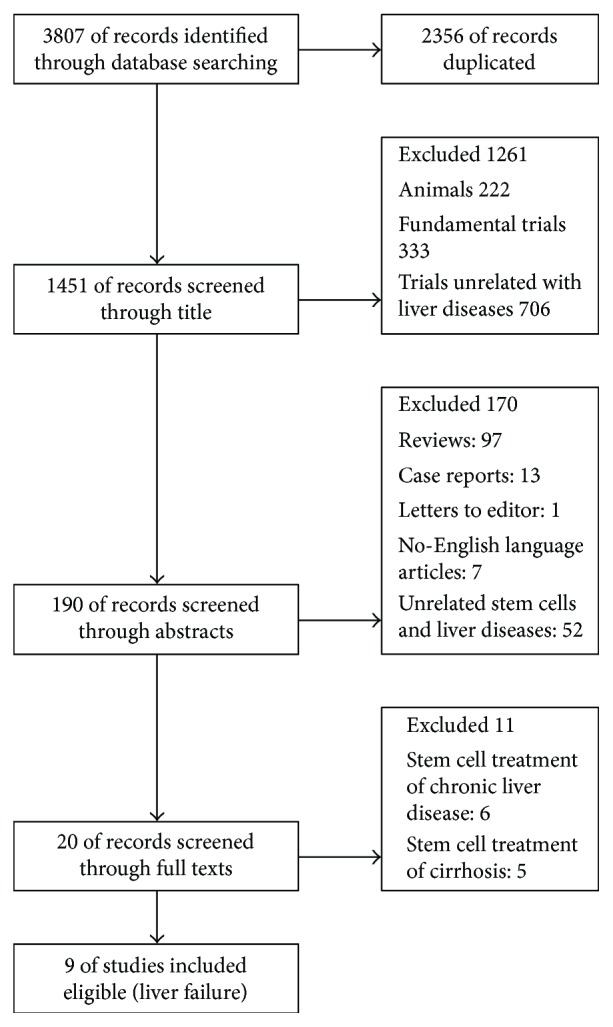
Flow diagram of included and excluded studies in this review.

A total of 1451 articles were initially identified with duplicate removal. Therefore, 20 articles were associated with MSCT in the treatment of liver disease through retrieval and evaluation in detail; of these, six trials were related with chronic liver disease [12, 25–29], five were involved with cirrhosis [30–34], and nine focused only on LF [1, 9, 16–18, 35–38] (Figure 1). All eligible studies' demographic and clinical characteristics of LF patients were summarized in Table 1 and Supplement Table 1. Among them, five trials belonged to the randomized controlled trials (RCTs) [16, 18, 35, 37, 38] and others were cohorts [1, 9, 17, 36]. A total of 463 patients were enrolled without missing the number of 14 patients, of which, 158 patients accepted the MSCT as MSC group and 305 patients of conventional therapy as control group. Articles enrolled in this review were conducted in Egypt, Korea, India, and China.

The nine studies enrolled had a total score of 63 with a mean of 7 and a range of 4 to 9 for each article based on NOS scoring system. All studies enrolled fallen into “high-quality study” (those of ≥4 scores). Overall, the quality of included studies was deemed eligible. The qualities of each study included in our review were showed in Table 2.

### 3.2. The Improvement of ALT, TBIL, ALB, and PT after MSCT

The liver function indexes were evaluated in our review, mainly including ALT, TBIL, ALB, and PT. There were six articles reporting on ALT with a total of 265 patients [1, 9, 16, 18, 36, 37], five on TBIL [16–18, 36, 37], seven on ALB [1, 9, 16, 18, 35–37], and three on PT [16, 18, 36]. But only one article [16] involved in AST, and its variations were showed in Table 3. We thus closely analyzed the variations of ALT, TBIL, ALB, and PT in the MSC group and control group at different time points. Meanwhile, we also compared the differences between the MSC group and control group at a certain time point.

**Figure 2:**
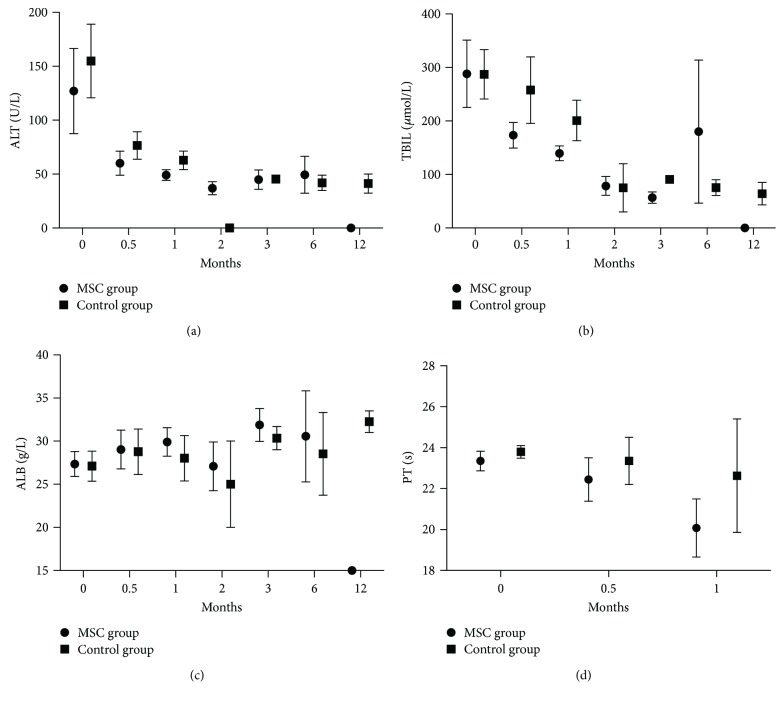
The improvement of ALT, TBIL, ALB, and PT between MSC group and control group. After MSCT, (a) the ALT baseline decreased in half a month (78.57 ± 30.23 versus 288.29 ± 140.54 *μ*mol/L, *P* < 0.05); (b) the TIBL baseline diminished largely at 2 and 3 months (56.74 ± 18.40 versus 288.29 ± 140.54 *μ*mol/L, *P* < 0.05); (c, d) the variations of ALB and PT at different time points had no statistical differences.

Among the patients in the MSC group, the ALT baseline of LF patients decreased from 127.02 ± 96.71 to 60.11 ± 22.36 U/L at half a month after MSCT, which had a significant statistical difference (*P* < 0.05); the levels were 49.16 ± 11.12, 36.98 ± 10.42, 44.98 ± 17.97, and 49.4 ± 24.18 U/L at 1, 2, 3, and 6 months, separately (Table 3, Figure 2(a)). As for the variations in TBIL, ALB, and PT, five of nine articles (*n* = 366) were related with TBIL [16–18, 36, 37], seven with ALB (*n* = 319) [1, 9, 16, 18, 35–37], and three with PT (*n* = 213) [18, 36]. Among them, the level of TBIL declined largely after MSCT at 2 and 3 months compared with the baseline (78.57 ± 30.23 versus 288.29 ± 140.54 *μ*mol/L, *P* < 0.05; 56.74 ± 18.40 versus 288.29 ± 140.54 *μ*mol/L, *P* < 0.05) (Table 3, Figure 2(b)). However, no significant differences were observed in obvious changes of ALB and PT at any time points (Table 3). Finally, there were no statistical differences in the control group and MSC group at each time point according to the variations of ALT, TBIL, ALB, and PT (Supplement Table 2 and Figures 2(a)–2(d)).

### 3.3. ACLF Group Had a Better Efficacy Compared with CLF Group Based on the *D* Value of MELD Scores after MSCT

**Figure 3:**
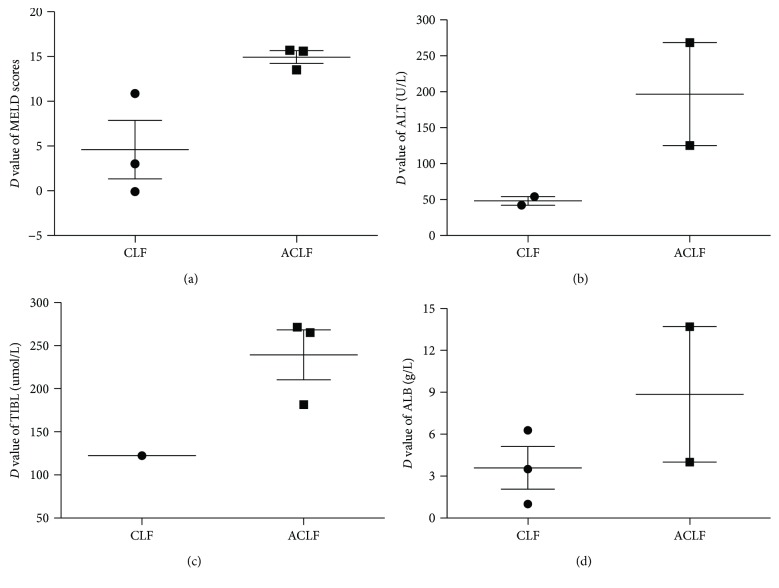
The variations of MELD scores, ALT, TIBL, and ALB between ACLF group and CLF group. (a) The *D* value of MELD score of ACLF group was higher than CLF group (14.93 ± 1.24 versus 4.6 ± 5.66, *P* < 0.05); (b, c, d) *D* values of ALT, TIBL, and ALB had no differences between CLF group and ACLF group, separately.

A total of six studies were enrolled, in which they mainly study the patients of CLF and ACLF [9, 16–18, 35, 37]. Our analysis thus divided LF patients into CLF group [9, 18, 35] and ACLF group [16, 17, 37]; of them, the *D* value of MELD score of the ACLF group was higher than that of the CLF group (14.93 ± 1.24 versus 4.6 ± 5.66, *P* < 0.05) (Figure 3(a)), while the *D* values of ALT, TIBL, and ALB had no difference between the CLF group and ACLF group (48.00 versus 196.7 U/L, 122.42 versus 226.43 *μ*mol/L, 3.59 versus 8.85 g/L) (Figures 3(b)–3(d)).

### 3.4. MELD Score Baseline of ≥20 Group Had Better Efficacy Compared with a Baseline of <20 Group after MSCT

**Figure 4:**
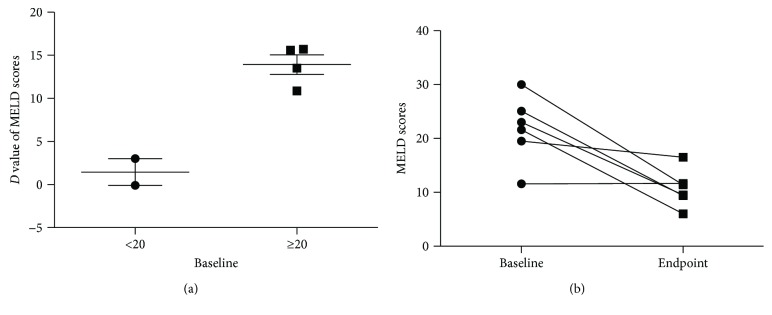
The level of MELD scores in LF. (a) MELD score baseline of ≥20 group had better efficacy compared with baseline of <20 group after MSCT; (b) the scores of MELD endpoint were concentrated in 10 points.

All six studies with a total of 400 cases were included [9, 16–18, 35, 37]. MELD score is calculated as this: 9.5^∗^In [creatinine (9 mg/dL)] + 3.78^∗^In [bilirubin (mg/dL)] + 11.2^∗^In (INR) + 6.43, which ranged from 6 (mild disease) to 40 (severe disease) [39]. All included studies were chiefly divided into MELD baseline of ≥20 group and MELD baseline of <20 group due to they concentrated in ≥20 points and <20 points. There was a significant statistical difference that MELD baseline of ≥20 group has a higher *D* value of MELD of 13.92 ± 2.27 compared with MELD baseline of <20 group (1.46 ± 2.18) after MSCT (*P* = 0.003) (Figure 4(a)). But beyond that, the scores of MELD endpoint were concentrated in 10 points, which was by chance a lower mortality rate was 1.9% at 3 months after MSCT [40] for LF patients with a score of 10 points (Figure 4(b)).

### 3.5. The Survival of LF Patients

**Figure 5:**
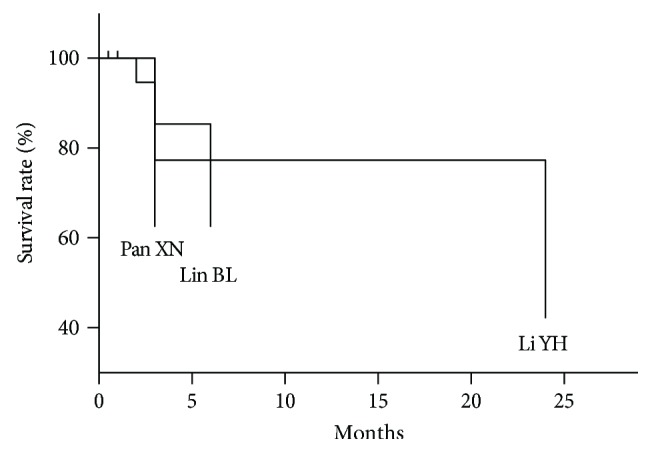
The survival of LF.

Only three studies are involved in the survival of LF patients in treatment of MSCT. Lin et al. [17] showed a higher survival rate of 85.34% at 3 months and of 62.48% at 6 months. Li et al. [16] had a longer follow-up time of 24 months, companied by a survival rate of 42.14%. All data were showed in Figure 5.

## 4. Discussion

MSC transplantation has been utilized gradually in clinical practice and been emerged as a novel intervention for the treatment of liver cell failure. And some articles demonstrated its advantages deeply and explored a systematic protocol [41, 42]. Of note is what we found in our review: (1) after MSCT, the level of ALT baseline declined largely in half a month, and the TBIL baseline declined at 2 and 3 months. Thereafter, both of them maintained at a steady level, which was considered as early evaluations of efficacy in treatment of LF after MSCT; (2) as shown in Figures 2(a)–2(d), MSCT had a comparable curative effect compared with conventional therapy in patients of liver cell failure in terms of ALT, TBIL, ALB, and PT; (3) the MSCT usage of ACLF had a more advantage than that of CLF; (4) the higher the MELD baseline (baseline of ≥20) in LF, the more efficacy of MSCT. They were benefited for further understanding and providing the rationale for improved disease management strategies.

The improvement of liver function was found after MSC treatment in a short time of less than 3 months, especially ALT (in half a month), which might be closely linked with the mechanisms of MSCs in the treatment of patients with LF. Wang et al. [2] hypothesized that MSCs could promote hepatocyte proliferation to stimulate liver regeneration; on the other hand, it differentiated into the parenchymal hepatocytes to improve the liver function [43]. However, other previous studies revealed that it was via secreting protective factors (hepatocyte growth factor (HGF) and epidermal growth factor (EGF)) that structured a well-done microenvironment to prevent aggressive damage [43–46]. Moreover, immunomodulation and antifibrosis of MSCs may play an important role in liver regeneration and delaying the liver cell progressive damage by downregulation of the level of liver fibrosis marker in liver cell failure [12, 47]. Our results showed that in 0.5 to 3 months after MSCT, the efficacy of mesenchymal stem cells was performed. Terai et al. [48] showed that liver cells repopulated 25% of the recipient's damaged liver by one month after MSCT at the model of mice with LF, which was supplementary of our results. Then, our other new finding was that MSCT had a more dominant advantage on ACLF than on CLF. Firstly, the hepatocytes have lively reverse-differentiate into stem cell to take participation in the regeneration of liver cells, while the hepatocytes of CLF patients almost lost their secretory and differentiation capacity (e.g., heme oxygenase-1) [49]. In addition, many inflammatory cells of T-lymphocyte and B-lymphocyte were involved in the acute inflammation activity of ACLF, which could be repressed by MSCs characterized by its anti-inflammatory ability [50–52]. Therefore, in the abovementioned statements, the mesenchymal stem cells had the ability of improving liver function and promoting liver regeneration.

MELD score was an objective assessment of patients with liver disease and was calculated by using a combination of blood tests: creatinine, serum bilirubin, and INR, whereas it lacks the information of portal hypertension [53]. And the Child-Pugh score offsets a lack of MELD score, which was originally designed for assessing the prognosis of patients with cirrhosis undergoing surgical treatment of portal hypertension. It used five parameters: total bilirubin, serum albumin, INR, ascites, and encephalopathy [35, 53]. Our analysis accumulated both of the data of MELD score and Child-Pugh score. But only three studies reported the information of the Child-Pugh score [1, 9, 35]. Park et al. showed improvement of the Child-Pugh score in two of five patients; at the same time, Amer supplemented that a statistically significant improvement appeared after 2 weeks and maintained for 6 months. However, due to the limited articles included, we cannot gain a definite conclusion about the Child-Pugh score. In contrast, the researches of MELD score among clinical trials were relatively mature. There was accumulating evidence that MELD score dramatically diminished after MSC therapy, especially in MELD baseline of ≥20 group. It will provide the evidence of the optimal state of MSCs in clinical practice.

There were several limitations. Firstly, the number of cases included in this review is small and the published works may not have covered all relevant references. Secondly, we are lack of the overall data of the type of cell—adipose-derived MSC, umbilical cord-derived MSC, and bone marrow-derived MSC; we thus could not compare their differences in treatment of liver diseases. Thirdly, there are two studies enrolled of less than five cases and evidence might be weak. Finally, almost no information on clinical symptoms (e.g., ascites, jaundice, and hemorrhage) was provided in the studies we included.

## 5. Conclusion

Our study analyzed the improvement of liver functions (ALT, TIBL, ALB, and PT) after MSCT and the impact of MSCT on MELD score characterized by immune tolerance of stem cells [54]. All of them can provide a systematic review of MSC application in LF patients. The results from the upcoming and ongoing preclinical and clinical trials will provide a valuable roadmap for these novel therapeutic options of MSCs that have the ability to successfully promote liver cell failure, and our results also provide a large value for clinical physicians and investigators in the future.

## Figures and Tables

**Table 1:** Demographic and clinical features at enrollment in clinical trials.

Source	Year	Country	Number of enrolled patients	Age (year)	Disease	Causes of disease	Type of cells	Route of administration	Follow-up (month)	Assessments
Pan et al.	2008	China	10	18–27	LF	NA	BMSC	Hepatic/splenic artery	3	ALT, AST, PT, TBIL, DBIL, ALB, fibrinogen
Khan et al.	2008	India	4	NA	CLF	HBV:1, HCV:3	BMMSC	Hepatic artery	NA	ALB, BILALT, Child score, MELD
Peng et al.	2011	China	BMSC:53, control:105	BMSC: 42.19 ± 10.8; control: 42.22 ± 11.37	CLF	HBV	BMMSC	Hepatic artery	48	ALT, TBIL, PT, ALB, MELD
Amer et al.	2011	Egypt	BMSC: 20, control: 20	BMSC: 50.5 ± 4.1, control: 55 ± 3.6	CLF	HCV	BMHC	NA	6	Child score, MELD
Shi et al.	2012	China	UCMSC: 24, control: 19	UCMSC: 40, control: 45	ACLF	HBV	UCMSC	Cubital vein of the arm	18	ALT, TBIL, ALB, CHE, PTA, MELD
Park et al.	2013	Korea	5	44 ± 7.07	LF	HBV: 2, HCV: 2, other 1	BMMNC	Hepatic artery	12	ALT, Cr, INR, CT, Child scores, QoL
Wan et al.	2013	China	ACLF: 30, control: 20	ACLF: 43, control: 39	ACLF	HBV	HSC	NA	NA	ALT, TIBL, Cr, IL-6, MMP-2/9, Ishak score, Child class, MELD
Li et al.	2016	China	UCMSC + PE: 11, PE: 34	UCMSC + PE: 51.1 ± 11.2, PE: 50.0 ± 10.9	ACLF	HBV	UCMSC	Hepatic artery	24	ALT, AST, DBIL, TBIL, Cr, DBIL, PT, INR, AFP, MELD, USG, CT
Lin et al.	2017	China	BMSC: 56, control: 54	BMMSC: 40.04 ± 9.94, control: 42.78 ± 8.40	ACLF	HBV	BMMSC	Peripheral veins	6	ALB, ALT, TBIL, INR, CT, MRI, US, Cr, MELD

Data are expressed as mean ± standard deviation. NA: not available; ACLF: acute-on-chronic liver failure; UCMSC: umbilical cord-derived mesenchymal stem cell; BMSC: bone marrow-derived mesenchymal stromal cell; HSC: hematopoietic stem cell; ALB: albumin; ALT: alanine aminotransferase; AST: aspartate transaminase; TBIL: total bilirubin; DBIL: direct bilirubin; PT: prothrombin time; INR: international normalized ratio; Cr: creatinine; MELD: model for end-stage liver disease score; QoL: quality of life; CT: computed tomography scan: MRI: magnetic resonance imaging; US: ultrasonography.

**Table 2:** Quality assessment of studies enrolled in liver cell failure.

Author	Year	Representativeness of the exposed cohort	Selection of the nonexposed cohort	Ascertainment of exposure	No demonstration of interesting outcome at start of study	Control for important factor or additional factor	Assessment of outcome	Enough follow-up of outcome	Adequacy of follow-up of cohorts	Total quality scores
Pan et al.	2008	1	0	1	1	2	1	1	1	8
Khan et al.	2008	1	0	1	1	1	0	0	0	4
Peng et al.	2011	1	1	1	1	2	0	0	1	7
Amer et al.	2011	1	1	1	1	2	0	1	1	8
Shi et al.	2012	1	1	1	1	2	1	1	1	9
Park et al.	2013	1	0	1	1	1	0	1	1	6
Wan et al.	2013	1	1	1	1	1	0	0	0	5
Li et al.	2016	1	1	1	1	1	1	1	1	8
Lin et al.	2017	1	0	1	1	2	1	1	1	8

Note: total score, 9; ≤4, poor quality; >4, good quality.

**Table 3:** The change of liver functions index after MSCT therapy.

Index	Follow-up of MSC group (month)							
	Baseline (0)	0.5	1	2	3	6	12	24
ALT (U/L)	127.02 ± 96.71^∗^a	60.11 ± 22.36^∗^b	49.16 ± 11.12	36.98 ± 10.42	44.98 ± 17.97	49.4 ± 24.18	NA	NA
AST (U/L)	232.4 ± 180.9	77.6 ± 10.3	71.6 ± 15.0	NA	85.0 ± 72.0	43.3 ± 19.6	35.0 ± 10.0	36.7 ± 9.6
TBIL (*μ*mol/L)	288.29 ± 140.54^∗^c	173.40 ± 41.38	139.53 ± 30.91	78.57 ± 30.23^∗^d	56.74 ± 18.40^∗^e	180.19 ± 188.92	NA	NA
ALB (g/L)	27.35 ± 3.85	29.03 ± 4.5	29.92 ± 4.06	27.08 ± 4.89	31.88 ± 3.79	30.57 ± 9.16	NA	NA
PT (s)	23.35 ± 0.83	22.44 ± 1.83	20.08 ± 2.45	NA	NA	NA	NA	NA

Data are expressed as mean ± standard deviation. NA: not available; ^∗^a, ^∗^b, ^∗^c, ^∗^d, and ^∗^e: *P* < 0.05.

## Supplementary Materials

Supplementary Materials Supplementary Table 1: supplementary demographic and clinical features at enrollment in clinical trials. Supplementary Table 2: the variations of liver functions among the patients in the control group. The level of ALT, AST, TBIL, ALB, and PT at the time of 0–24 months among the patients in the control group.

## References

[1] Park C. H., Bae S. H., Kim H. Y. (2013). A pilot study of autologous CD34-depleted bone marrow mononuclear cell transplantation via the hepatic artery in five patients with liver failure. *Cytotherapy*.

[2] Wang K., Chen X., Ren J. (2015). Autologous bone marrow stem cell transplantation in patients with liver failure: a meta-analytic review. *Stem Cells and Development*.

[3] Kayaalp C., Ersan V., Yilmaz S. (2014). Acute liver failure in Turkey: a systematic review. *The Turkish Journal of Gastroenterology*.

[4] Sarin S. K., Kedarisetty C. K., Abbas Z. (2014). Acute-on-chronic liver failure: consensus recommendations of the Asian Pacific Association for the Study of the Liver (APASL) 2014. *Hepatology International*.

[5] Abbas Z., Shazi L. (2015). Pattern and profile of chronic liver disease in acute on chronic liver failure. *Hepatology International*.

[6] Blachier M., Leleu H., Peck-Radosavljevic M., Valla D. C., Roudot-Thoraval F. (2013). The burden of liver disease in Europe: a review of available epidemiological data. *Journal of Hepatology*.

[7] Streetz K. L., Tacke F., Koch A., Trautwein C. (2013). Akutes Leberversagen. *Medizinische Klinik - Intensivmedizin und Notfallmedizin*.

[8] Sarin S. K., Choudhury A. (2016). Acute-on-chronic liver failure: terminology, mechanisms and management. *Nature Reviews Gastroenterology & Hepatology*.

[9] Khan A. A., Parveen N., Mahaboob V. S. (2008). Safety and efficacy of autologous bone marrow stem cell transplantation through hepatic artery for the treatment of chronic liver failure: a preliminary study. *Transplantation Proceedings*.

[10] Theise N. D. (2003). Liver stem cells. *Cytotechnology*.

[11] Cao Y., Ding Z., Han C., Shi H., Cui L., Lin R. (2017). Efficacy of mesenchymal stromal cells for fistula treatment of Crohn’s disease: a systematic review and meta-analysis. *Digestive Diseases and Sciences*.

[12] Salama H., Zekri A. R., Medhat E. (2014). Peripheral vein infusion of autologous mesenchymal stem cells in Egyptian hcv-positive patients with end-stage liver disease. *Stem Cell Research & Therapy*.

[13] Akhurst B., Matthews V., Husk K., Smyth M. J., Abraham L. J., Yeoh G. C. (2005). Differential lymphotoxin-β and interferon gamma signaling during mouse liver regeneration induced by chronic and acute injury. *Hepatology*.

[14] Amiri F., Molaei S., Bahadori M. (2016). Autophagy-modulated human bone marrow-derived mesenchymal stem cells accelerate liver restoration in mouse models of acute liver failure. *Iranian Biomedical Journal*.

[15] Banas A., Teratani T., Yamamoto Y. (2009). Rapid hepatic fate specification of adipose-derived stem cells and their therapeutic potential for liver failure. *Journal of Gastroenterology and Hepatology*.

[16] Li Y. H., Xu Y., Wu H. M., Yang J., Yang L. H., Yue-Meng W. (2016). Umbilical cord-derived mesenchymal stem cell transplantation in hepatitis B virus related acute-on-chronic liver failure treated with plasma exchange and entecavir: a 24-month prospective study. *Stem Cell Reviews and Reports*.

[17] Lin B. L., Chen J. F., Qiu W. H. (2017). Allogeneic bone marrow-derived mesenchymal stromal cells for hepatitis B virus-related acute-on-chronic liver failure: a randomized controlled trial. *Hepatology*.

[18] Peng L., Xie D. Y., Lin B. L. (2011). Autologous bone marrow mesenchymal stem cell transplantation in liver failure patients caused by hepatitis B: short-term and long-term outcomes. *Hepatology*.

[19] Lin R., Ding Z., Ma H. (2015). In vitro conditioned bone marrow-derived mesenchymal stem cells promote de novo functional enteric nerve regeneration, but not through direct-transdifferentiation. *Stem Cells*.

[20] Lin R., Ma H., Ding Z. (2013). Bone marrow-derived mesenchymal stem cells favor the immunosuppressive T cells skewing in a *Helicobacter pylori* model of gastric cancer. *Stem Cells and Development*.

[21] Okumoto K., Saito T., Onodera M. (2007). Serum levels of stem cell factor and thrombopoietin are markedly decreased in fulminant hepatic failure patients with a poor prognosis. *Journal of Gastroenterology and Hepatology*.

[22] Mirotsou M., Jayawardena T. M., Schmeckpeper J., Gnecchi M., Dzau V. J. (2011). Paracrine mechanisms of stem cell reparative and regenerative actions in the heart. *Journal of Molecular and Cellular Cardiology*.

[23] Ownby R. L., Crocco E., Acevedo A., John V., Loewenstein D. (2006). Depression and risk for Alzheimer disease: systematic review, meta-analysis, and metaregression analysis. *Archives of General Psychiatry*.

[24] Lin R., Murtazina R., Cha B. (2011). D-Glucose acts via sodium/glucose cotransporter 1 to increase NHE3 in mouse jejunal brush border by a Na+/H+ exchange regulatory factor 2-dependent process. *Gastroenterology*.

[25] Andreone P., Catani L., Margini C. (2015). Reinfusion of highly purified CD133^+^ bone marrow-derived stem/progenitor cells in patients with end-stage liver disease: a phase I clinical trial. *Digestive and Liver Disease*.

[26] Huang X. L., Luo L., Luo L. Y. (2014). Clinical outcome of autologous hematopoietic stem cell infusion via hepatic artery or portal vein in patients with end-stage liver diseases. *Chinese Medical Sciences Journal*.

[27] Levicar N., Pai M., Habib N. A. (2008). Long-term clinical results of autologous infusion of mobilized adult bone marrow derived CD34^+^ cells in patients with chronic liver disease. *Cell Proliferation*.

[28] Salama H., Zekri A. R., Bahnassy A. A. (2010). Autologous CD34^+^ and CD133^+^ stem cells transplantation in patients with end stage liver disease. *World Journal of Gastroenterology*.

[29] Salama H., Zekri A. R., Zern M. (2010). Autologous hematopoietic stem cell transplantation in 48 patients with end-stage chronic liver diseases. *Cell Transplantation*.

[30] Cai T., Deng Q., Zhang S., Hu A., Gong Q., Zhang X. (2015). Peripheral blood stem cell transplantation improves liver functional reserve. *Medical Science Monitor*.

[31] Mohamadnejad M., Alimoghaddam K., Bagheri M. (2013). Randomized placebo-controlled trial of mesenchymal stem cell transplantation in decompensated cirrhosis. *Liver International*.

[32] Mohamadnejad M., Namiri M., Bagheri M. (2007). Phase 1 human trial of autologous bone marrow-hematopoietic stem cell transplantation in patients with decompensated cirrhosis. *World Journal of Gastroenterology*.

[33] Mohamadnejad M., Vosough M., Moossavi S. (2016). Intraportal infusion of bone marrow mononuclear or CD133^+^ cells in patients with decompensated cirrhosis: a double-blind randomized controlled trial. *Stem Cells Translational Medicine*.

[34] Nikeghbalian S., Pournasr B., Aghdami N. (2011). Autologous transplantation of bone marrow-derived mononuclear and CD133^+^ cells in patients with decompensated cirrhosis. *Archives of Iranian Medicine*.

[35] Amer M. E., El-Sayed S. Z., El-Kheir W. A. (2011). Clinical and laboratory evaluation of patients with end-stage liver cell failure injected with bone marrow-derived hepatocyte-like cells. *European Journal of Gastroenterology & Hepatology*.

[36] Pan X. N., Shen J. K., Zhuang Y. P. (2008). Autologous bone marrow stem cell transplantation for treatment terminal liver diseases. *Journal of Southern Medical University*.

[37] Shi M., Zhang Z., Xu R. (2012). Human mesenchymal stem cell transfusion is safe and improves liver function in acute-on-chronic liver failure patients. *Stem Cells Translational Medicine*.

[38] Wan Z., You S., Rong Y. (2013). CD34+ hematopoietic stem cells mobilization, paralleled with multiple cytokines elevated in patients with HBV-related acute-on-chronic liver failure. *Digestive Diseases and Sciences*.

[39] Singal A. K., Kamath P. S. (2013). Model for end-stage liver disease. *Journal of Clinical and Experimental Hepatology*.

[40] Wiesner R., Edwards E., Freeman R. (2003). Model for end-stage liver disease (MELD) and allocation of donor livers. *Gastroenterology*.

[41] Viswanathan P., Gupta S. (2012). New directions for cell-based therapies in acute liver failure. *Journal of Hepatology*.

[42] Than N. N., Tomlinson C. L., Haldar D., King A. L., Moore D., Newsome P. N. (2016). Clinical effectiveness of cell therapies in patients with chronic liver disease and acute-on-chronic liver failure: a systematic review protocol. *Systematic Reviews*.

[43] Houlihan D. D., Newsome P. N. (2008). Critical review of clinical trials of bone marrow stem cells in liver disease. *Gastroenterology*.

[44] Kieling C. O., Uribe-Cruz C., Lopez M. L., Osvaldt A. B., da Silveira T. R., Matte U. (2017). Paracrine effects of bone marrow mononuclear cells in survival and cytokine expression after 90% partial hepatectomy. *Stem Cells International*.

[45] Germain L., Noel M., Gourdeau H., Marceau N. (1988). Promotion of growth and differentiation of rat ductular oval cells in primary culture. *Cancer Research*.

[46] Oh S. H., Miyazaki M., Kouchi H. (2000). Hepatocyte growth factor induces differentiation of adult rat bone marrow cells into a hepatocyte lineage *in vitro*. *Biochemical and Biophysical Research Communications*.

[47] Esrefoglu M. (2013). Role of stem cells in repair of liver injury: experimental and clinical benefit of transferred stem cells on liver failure. *World Journal of Gastroenterology*.

[48] Terai S., Sakaida I., Yamamoto N. (2003). An *in vivo* model for monitoring *trans*-differentiation of bone marrow cells into functional hepatocytes. *Journal of Biochemistry*.

[49] Zhang Z. H., Zhu W., Ren H. Z. (2017). Mesenchymal stem cells increase expression of heme oxygenase-1 leading to anti-inflammatory activity in treatment of acute liver failure. *Stem Cell Research & Therapy*.

[50] Liang C., Jiang E., Yao J. (2017). Interferon-γ mediates the immunosuppression of bone marrow mesenchymal stem cells on T-lymphocytes *in vitro*. *Hematology*.

[51] Ma X., Che N., Gu Z. (2013). Allogenic mesenchymal stem cell transplantation ameliorates nephritis in lupus mice via inhibition of B-cell activation. *Cell Transplantation*.

[52] Pontikoglou C., Kastrinaki M. C., Klaus M. (2013). Study of the quantitative, functional, cytogenetic, and immunoregulatory properties of bone marrow mesenchymal stem cells in patients with B-cell chronic lymphocytic leukemia. *Stem Cells and Development*.

[53] Angermayr B., Cejna M., Karnel F. (2003). Child-Pugh versus MELD score in predicting survival in patients undergoing transjugular intrahepatic portosystemic shunt. *Gut*.

[54] Khubutiya M., Temnov A. A., Vagabov V. A., Sklifas A. N., Rogov K. A., Zhgutov Y. A. (2015). Effect of conditioned medium and bone marrow stem cell lysate on the course of acetaminophen-induced liver failure. *Bulletin of Experimental Biology and Medicine*.

