# Supplementary material for: Mesenchymal Stem Cell Transplantation for Liver Cell Failure: A New Direction and Option
Source: Gastroenterol Res Pract. 2018 Mar 4;2018:9231710. doi: 10.1155/2018/9231710 (PMC5857323; doi:10.1155/2018/9231710)
Supplement: Supplementary Materials — Supplementary Table 1: supplementary demographic and clinical features at enrollment in clinical trials. Supplementary Table 2: the variations of liver functions among the patients in the control group. The level of ALT, AST, TBIL, ALB, and PT at the time of 0–24 months among the patients in the control group. [file 9231710.f1.docx]

Supplement table 1 Demographic and clinical features at enrollment in clinical trials

| Source | Year | Country | RCT | Age(mean±SD, range) | Male/total | Sources of cells |
| --- | --- | --- | --- | --- | --- | --- |
| Pan XN | 2008 | China | No | 18-27 | NA | Autologous |
| Khan AA | 2008 | India | No | NA | NA | Autologous |
| Peng L | 2011 | China | Yes | BMSC:42.19±10.8 ;control:42.22 ±11.37 | BMSC:50/53 ;control:99/105 | Autologous |
| Amer ME | 2011 | Eygpt | Yes | BMMSC:50.5 ±4.1 ,control:55±3.6 | BMMSC:16/20,control:17/20 | Autologous |
| Shi M | 2012 | China | Yes | UCMSC:40,control:45 | UCMSC:20/24,control:15/19 | NA |
| Park CH | 2013 | Korea | No | 44±7.07 | 2/5 | Autologous |
| Wan Z | 2013 | China | Yes | ACLF:43,control：39 | ACLF:22/30, control：14/20 | NA |
| Li YH | 2016 | China | Yes | UCMSC+PE:51.1 ± 11.2 ,PE:50.0 ± 10.9 | UCMSC+PE:8/11,PE:26/34 | NA |
| Lin BL | 2017 | China | No | BMMSC:40.04±9.94,control:42.78±8.40 | BMMSC:51/56,control:53/54 | Allogeneic |

Supplement table 2 The variations of liver functions among control group

| Index | Follow-up of control group（month） | | | | | | | |
| --- | --- | --- | --- | --- | --- | --- | --- | --- |
|  | Baseline（0） | 0.5 | 1 | 2 | 3 | 6 | 12 | 24 |
| ALT(U/L) | 154.89±59.22 | 76.51±22.2 | 62.86±14.78 | NA | 45.35±5.16 | 41.85±10.11 | 41.2±12.45 | NA |
| AST(U/L) | 200.9 ± 128.6 | 88.1 ± 14.5 | 83.0 ± 12.3 | NA | 60.8 ± 19.5 | 52.5 ± 11.5 | 50.4 ± 14.8 | 41.0 ± 9.2 |
| TBIL(umol/L) | 287.17±79.96 | 257.76±107.6 | 200．95±65.55 | 75±63.64 | 90.75±1.06 | 75.45±20.58 | 64.1±29.56 | NA |
| ALB(g/L) | 27.11±3.49 | 28.78±5.27 | 28.02±5.28 | 25±7.07 | 30.35±1.91 | 28.53±8.29 | 32.25±1.77 | NA |
| PT(s) | 23.80±0.43 | 23.35±1.63 | 22.63±3.91 | NA | NA | NA | NA | NA |
